# Do we need to suture the pronator quadratus muscle when we do open reduction and internal fixation for fracture of the distal radius

**DOI:** 10.1186/s12891-020-03450-8

**Published:** 2020-07-11

**Authors:** Kaibin Fang, Xiaocong Lin, Xiaolin Liu, Qingfeng Ke, Shaoojian Shi, Zhangsheng Dai

**Affiliations:** Department of Orthopaedic Surgery, The Second Affiliated Hospital of Fujian Medical University, No.34, Zhongshanbeilu, Quanzhou, 36200 Fujian China

**Keywords:** PQ muscle, Distal radius fracture, Open reduction and internal fixation

## Abstract

**Background:**

Open reduction and internal fixation is often used for the treatment of distal radius fracture. Opening the pronator quadratus muscle during the process of open reduction and internal fixation is necessary to achieve sufficient exposure. Therefore, knowledge on how to suture the pronator quadratus muscle will be of essence.

**Aim:**

The aim of the present study was to determine if suturing the pronator quadratus during the treatment of the distal radius fracture can enhance limb function .

**Methods:**

A total of 126 patients were enrolled for the study. The patients underwent open reduction and internal fixation. During the procedure, the pronator quadratus was cut open to allow insertion of the plate. The pronator quadratus muscles of the patients were stitched together before the surgery was completed. After the fracture healed, the patients underwent surgery to remove the internal fixations. Patients received wrist function scores prior to removal of the internal fixations. Healing of the pronator quadratus was during surgery. Patients were grouped according to the healing of the pronator quadratus. Functional scores between the two groups were compared.

**Results:**

Muscle healing was observed in 23 patients during surgery. However, the PQ muscles of these patients were remarkably atrophic, with scar hyperplasia and fibrosis. The muscle fibers were loose, thin, and had decreased in number. The remaining muscle fibers presented different degrees of adhesion with radial carpal flexor muscles, steel plates and interosseous membrane. A total of 23 patients were included in group A and 103 patients in group B based on the intraoperative condition. No statistically significant differences was observed in age and type of fracture between group A and group B. In addition, no statistically significant differences was observed in the isokinetic forearm pronation strength and clinical outcomes including grip strength, wrist ROM, and PRWE scores between the two groups.

**Conclusion:**

This study demonstrates that healing of the PQ muscle does not affect the outcomes of volar plating for distal radius fractures with reference to the isokinetic forearm rotation strength, grip strength, wrist ROM, and PRWE scores. The results of this study support our current practice of PQ muscle incision.

## Background

Pronator quadratus (PQ) muscle is a quadrilateral muscle located on the palmar side of the distal forearm, which is attached to the interosseous membrane of the radius, ulna and forearm. PQ comprises superficial and deep head. The deep head is thicker than the superficial head. The average volume of the whole muscle is 5.5 cm × 5.0 cm × 1.0 cm. The anterior interosseous artery and the anterior interosseous nerve are the primary artery and nerve of the pronator, respectively. The principal function of pronator is to rotate the forear m[1].

The PQ muscle is often sliced open during the treatment of distal radius fractures using open reduction and internal fixation with steel plates [2]. There are still controversies on the significance of PQ muscle repair [3, 4]. Most researchers adopted the method of prospective research in previous studies, which mainly divided patients into two groups: patients with repaired PQ muscles and patients without repaired PQ muscles. The efficacy between the two groups was subsequently compared [3–6]. However, the question of whether the anterior rotator cuff healed after repair was not addressed by the study. The effect of PQ muscle healing on wrist function remains unclear. This may be because most patients do not require removal of the internal fixation [7]. We removed the internal fixations of more than 100 patients with distal radius fractures who underwent open reduction and plate internal fixation. Patient groups were divided based on the healing of the PQ muscle identified during the operation. The wrist function of a patient was evaluated before the removal of the internal fixation. The aim of this study was to investigate the effect of PQ muscle healing on the wrist function of a patient.

## Methods

**Fig. 1:**
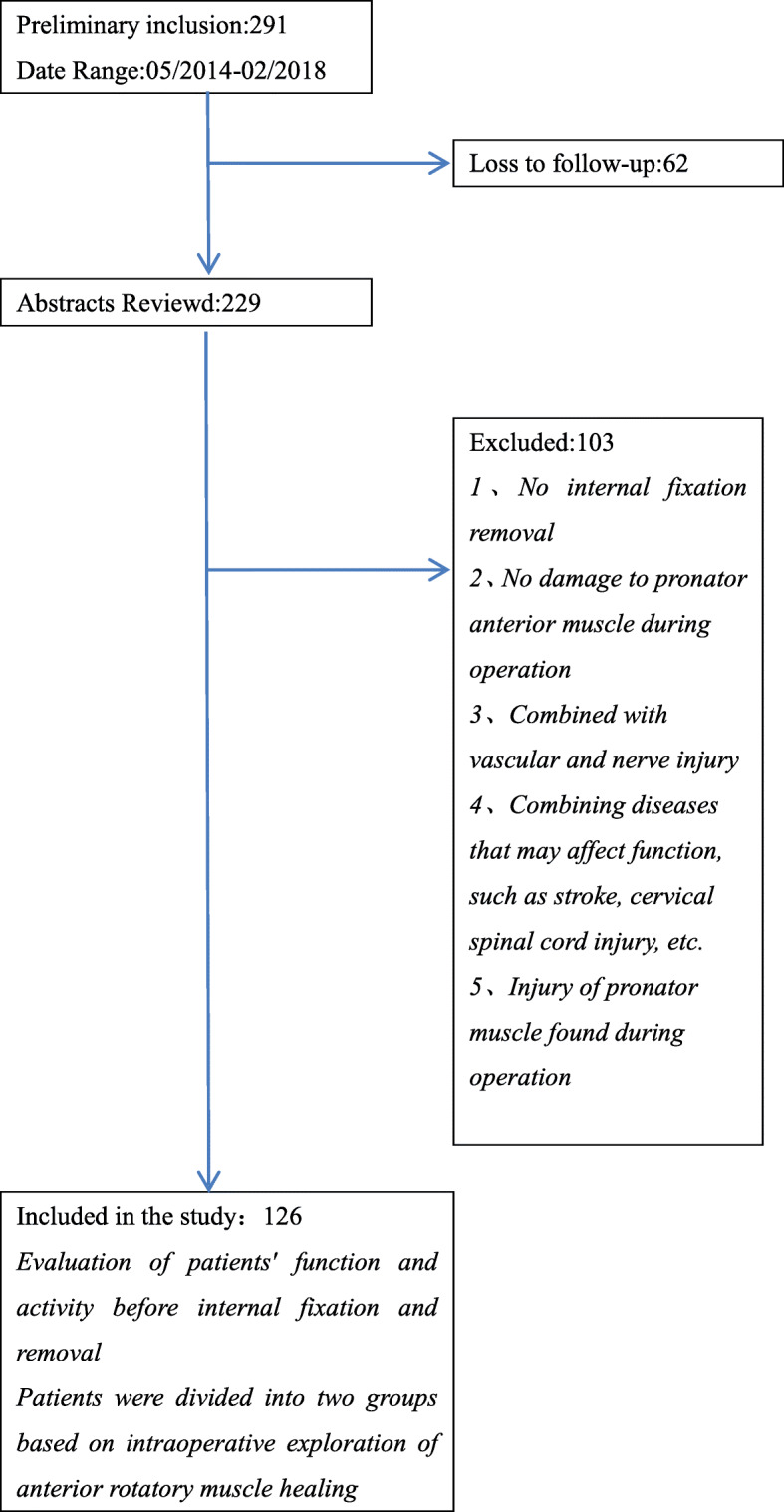
Inclusion and exclusion criteria

We conducted a retrospective study between May 2014 and February 2018. A total of 126 patients with distal radius fractures who underwent open reduction and plate internal fixation at the Second Affiliated Hospital of Fujian Medical University were enrolled for the study. Patients with distal radius fractures who met the inclusion criteria (age, > 18 years old) were included in the study. The radiographs of all initial fractures were reviewed and fractures classified as type A (extra-articular fractures), B (partial articular fractures), or C (complex articular fractures), and their subtypes according to the AO fracture classification. The current study was approved by the Hospital Ethics Committee of the Second Affiliated Hospital of Fujian Medical University (No.2020–183) (Fig. 1).

All surgical procedures were performed by 2 principal investigators. The chief surgeon, the corresponding author of this paper, had more than 20 years of experience in performing surgery thus qualified for this operation. The palmar, Henry approach was adopted in all surgeries. A section of the transverse carpal ligament was slit open and the radial artery and flexor tendon retracted to expose the PQ muscle. The PQ muscle was severed from the radial stop of the radius at 2–3 mm, and then detached above the periosteum. Finally, the muscle was pulled to the ulnar side to fully expose the metacarpal surface of the distal radius. Fractures were fixed using a volar locking plate system (VA-LCP Distal Radius Plate 2.4 mm; Synthes, Oberdorf, Switzerland). The PQ muscle was tightly stitched to the insertion using absorbable sutures (Ethicon, Johnson & Johnson, USA) with the forearm in supination at the end of the operation.

Patients visited the clinic 3–5 days after the operation to the examination of the surgical incision. Subsequent follow-up was conducted at 3 and 6 weeks, and 3 and 6 months. Afterwards, passive wrist motion below 90° of forward flexion was performed under the supervision of a therapist for the first 3 weeks. Patient exercises were progressed to full active motion as tolerated by each patient at 6 weeks after surgery. Patients were permitted to resume full activities and weight-bearing tolerable by the fracture after an X-ray confirmation of fracture healing.

**Fig. 2:**
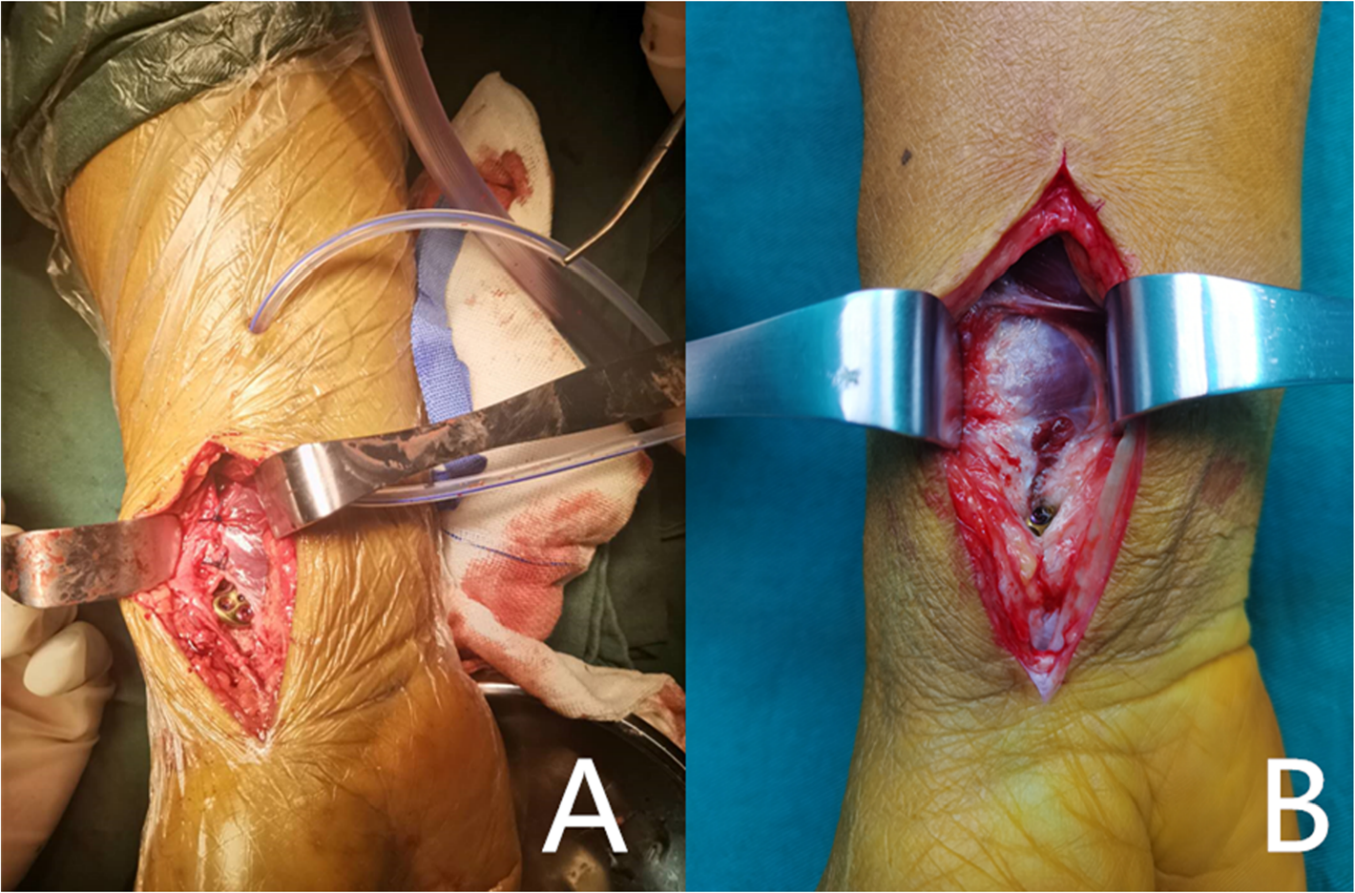
The patient's PQ muscle is considered healed

**Fig. 3:**
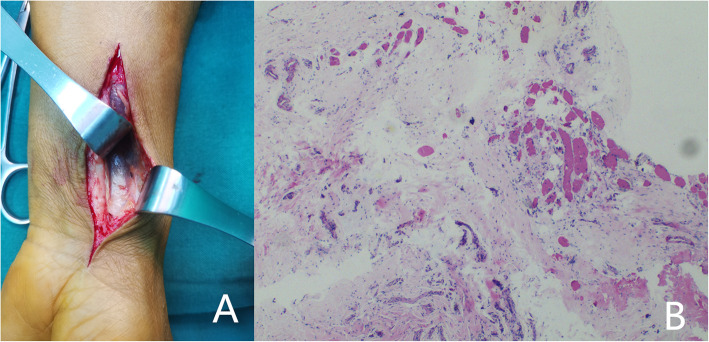
PQ muscle of the patient did not heal and was confirmed by pathology

The removal of the internal fixation was performed a year after the fracture had healed. Removal of the internal fixation primarily depended on a patient’s decision. Most patients opted to retain the internal fixations because they could develop localized pain symptoms or other complications if removed. The key reason for the removal of the internal fixation in patients was that they did not want to have a foreign body. Prior to the removal of the internal fixation, the wrist function and wrist motion of the patients were recorded. The Patient-Rated Wrist Evaluation (PRWE) score was used to evaluate the wrist function of patients. Henry’s approach to the original surgical incision was performed to remove the internal fixations. The soft tissue was separated between the flexor carpi radialis and the radial artery to expose the PQ muscle, followed by an assessment of the PQ muscle healing (Fig. 2). Unrecovered patients had a few or no muscle fibers and complete muscle scarring (Fig. 3). The PQ muscles of recovered patients were significantly atrophic with scar hyperplasia and fibrosis. The muscle fibers of the PQ muscles were loose, thin, and had decreased in number. We concluded that the PQ muscles of patients did not heal in the cases where PQ muscle fibres were not observed.

### Statistical analysis

We divided patients into two groups: the recovered group (group A) and the unrecovered group (group B) based on whether PQ muscles healed after removal of the internal fixation. The 2 groups were compared in terms of demographic data, isokinetic forearm rotation strength, and clinical outcomes including grip strength, wrist range of motion (ROM), and PRWE scores [8] before removal of the internal fixation. At a constant speed in the ROM to measure the isokinetic muscle strength [9]. The measurement of isokinetic muscle strength of the forearm rotation is an effective and reliable method to evaluate muscle function [10]. We used Biodex System 4 (Biodex Medical Systems, Shirley, NY, USA) to test at 90°/ s. We first assessed the uninjured side and then the injured side. The patient performed 10 repetitions of isokinetic motion for the measurement of peak torque (maximum torque measured in Newton-meters (Nm)) and total work (maximum amount of work performed in Joules (J)). The Exacta electronic dynamometer (nc70142-hkp; B & L engineering, USA) was used to measure the grip strength. The elbow was bent at 90° and the forearm rotated in a neutral direction during measurement of the grip strength. Grip strength values were expressed in kilograms [11]. Two doctors measured the wrist ROM of patients with a standard goniometer, and they remained blinded to the results of measurements. Patients were requested to complete the PRWE questionnaire [8], which evaluated the general disabilities related to the upper extremity. The PRWE scores ranged from 0 to100, where higher scores indicated less upper extremity of disability. Data for the categorical variables were analyzed using the chi-square test, whereas continuous data was analyzed using the t-test.

## Results

We found that the PQ muscle of most patients was replaced by scar, although the pronator muscle was sutured. Twenty-three patients were considered to have muscle healing during surgery. However, in these patients, the PQ muscles were obviously atrophic, with scar hyperplasia and fibrosis.The muscle fibers were loose and thin, and the number was reduced. At the same time, the remaining muscle fibers have different degrees of adhesion with radial carpal flexor muscles, steel plates and interosseous membrane. According to the intraoperative situation,23 patients were included in group A and 103 patients were included in group B.

There were no statistically significant differences in age(*p* = 0.22) and fracture type(*p* = 0.68) between group A and group B. At last follow-up,there were also no statistically significant differences in isokinetic forearm pronation strength(*p*>0.05), grip strength(*p* = 0.83), wrist range of motion and PRWE scores(*p* = 0.76) between the two groups. (Table 1).

**Table 1:** Demographic Data and Clinical Outcomes

Variable	Group A (*n* = 23)	Group B (*n* = 103)	Statistic (T-value /χ^2^-value)	*p*-value
Gender (Male / female)	(12/11)	(61/42)	0.38	0.54
Age (yr)	53.65 ± 8.63	55.85 ± 7.56	1.23	0.22
Affected hand (dominant /non-dominant)	(16/7)	(51/52)	3.03	0.08
Fracture type			0.77	0.68
A	9	17		
B	3	6		
C	29	80		
Isokinetic strength (%)^a^				
Peak pronation strength	79.23 ± 21.17	80.19 ± 19.27	0.21	0.83
Total pronation work	73.16 ± 31.65	72.36 ± 27.39	0.12	0.90
Peak supination strength	78.92 ± 27.16	79.25 ± 28.36	0.05	0.96
Total supination work	74.19 ± 33.02	73.53 ± 30.26	0.09	0.93
Grip strength (%)	71.36 ± 19.86	72.51 ± 23.52	0.22	0.83
Wrist range of motion (°)				
Flexion	53.33 ± 9.55	52.27 ± 10.69	0.44	0.66
Extension	68.75 ± 11.16	63.33 ± 15.52	1.58	0.12
Pronation	79.36 ± 10.73	77.55 ± 11.76	0.68	0.50
Supination	72.69 ± 13.12	73.67 ± 9.72	0.41	0.68
PRWE score	16.33 ± 15.69	17.56 ± 17.55	0.31	0.76

Values are presented as mean ± SD

*PRWE* Patient-Rated Wrist Evaluation

^a^For isokinetic strength, values after adjusting for hand dominance are presented in the parentheses

## Discussion

The use of volar locking plate to treat distal radius fractures has become increasingly popular over the last few years. Several studies have reported that patients achieve better functional scores after surgery with fewer complications [4–6]. However, controversy still surrounds intraoperative repair of the anterior rotator cuff.

Although the pronator of patients was repaired during the operation, the pronator strength was still affected [12]. This may be ascribed to factors such as the original muscle injury, loose suture, muscle tension change and muscle atrophy. A clinical case observation study by Swigart and colleagues revealed that the rate of failure of the PQ muscle repair was 4%, with 1 failure for every 24 patients. The study also revealed that the occurrence of preoperative injury to the PQ muscle was not associated with surgical failure [13]. In the study by Swigart et al., radiopaque hemoclips were attached to each side of the PQ repair. The patients underwent an X-ray re-examination after surgery to establish if the radiopaque hemoclips had shifted. Nevertheless, this study demonstrated that the incidence of repair success was lower than that previously reported by direct observation. The normal anatomical structure of PQ muscle is the basis of its function [14]. We also observed that the PQ muscles were all scarred, significantly atrophic, and adhered to the surrounding tissues after the anterior rotatory muscles were sutured. Muscle function is largely impaired because of the distortion of its anatomy. This may be attributed to the following factors: after the muscle is severed, blood supply to the muscle from the radial artery is disrupted, thus affecting repair of the muscles. The anterior rotator cuff is a brittle piece of muscle that is difficult to suture. This muscle frequently tears apart after stitching. The tension in the local soft tissues significantly increases especially after the insertion of a steel plate, making it difficult to pull the muscles together. In addition, the PQ muscle is prone to adhesion with surrounding tissues during the healing process, consequently affecting its function. The affected limb should not be weight bearing until the fracture heals because fractures take longer to heal. Disuse atrophy regularly occurs in the anterior rotatory muscle after a fracture heals.

The present study also revealed that healing of the pronator muscle did not enhance wrist function in patients. No significant differences were observed in the functional scores, activity levels, and grip strength between the two groups. Primary injury may not be a common cause of postoperative loss of muscle function because of the exclusion of patients with anterior rotatory muscle injury in this study. Nho [15] also established that the PQ muscle exhibited muscle atrophy during removal of the internal fixation, and that the muscle width was independent of the final clinical functional outcome. This suggests that function of the PQ muscle may be lost after it is sutured. Pronator teres is the primary muscle responsible for forearm pronation [16], thus its impairment can be resolved by pronator teres to maintain rotation of the forearm. Several studies have also indicated that suturing the pronator anterior muscle may not enhance wrist function and mobility in patients [17–19].

### Limitations

The present study had a few limitations. First, the sample size of our study was small. Second, this study did not evaluate the possible effects of combined injuries around the wrist on the rotational function of the wrist. Therefore, future studies should address the present limitations to validate the findings of the present study and to obtain more comprehensive results.

## Conclusion

This study demonstrates that the PQ muscle healing does not affect the outcomes of the volar plating for distal radius fractures in terms of isokinetic forearm rotation strength, grip strength, wrist ROM, and PRWE scores. The results of this study support our current practice of incising the PQ muscle. Most surgeons expose fractures by incising the PQ muscle, which shows that the repair of PQmuscle is not essential in enhancing forearm function.

## Data Availability

The datasets used and/or analyzed during current study are available from the corresponding author on reasonable request.

## Abbreviations

PQ muscle: Pronator quadratus muscle
PRWE: Patient-Rated Wrist Evaluation
